# Machine Learning and Radiomics Analysis for Tumor Budding Prediction in Colorectal Liver Metastases Magnetic Resonance Imaging Assessment

**DOI:** 10.3390/diagnostics14020152

**Published:** 2024-01-09

**Authors:** Vincenza Granata, Roberta Fusco, Maria Chiara Brunese, Gerardo Ferrara, Fabiana Tatangelo, Alessandro Ottaiano, Antonio Avallone, Vittorio Miele, Nicola Normanno, Francesco Izzo, Antonella Petrillo

**Affiliations:** 1 Division of Radiology, “Istituto Nazionale Tumori IRCCS Fondazione Pascale—IRCCS di Napoli”, 80131 Naples, Italy; a.petrillo@istitutotumori.na.it; 2 Medical Oncology Division, Igea SpA, 80013 Naples, Italy; r.fusco@igeamedical.com; 3 Department of Medicine and Health Sciences V. Tiberio, University of Molise, 86100 Campobasso, Italy; mariachiarabrunese@gmail.com; 4 Division of Pathology, “Istituto Nazionale Tumori IRCCS Fondazione Pascale—IRCCS di Napoli”, 80131 Naples, Italy; gerardo.ferrara@istitutotumori.na.it (G.F.); f.tatangelo@istitutotumori.na.it (F.T.); 5 Clinical Sperimental Abdominal Oncology Unit, Istituto Nazionale Tumori, IRCCS Fondazione G. Pascale, 80131 Naples, Italy; a.ottaiano@istitutotumori.na.it (A.O.); a.avallone@istitutotumori.na.it (A.A.); 6 Cell Biology and Biotherapy Unit, Istituto Nazionale Tumori IRCCS Fondazione Pascale—IRCCS di Napoli, 80131 Naples, Italy; vmiele@sirm.org; 7 Department of Radiology, University of Florence—Azienda Ospedaliero—Universitaria Careggi, 50134 Florence, Italy; n.normanno@istitutotumori.na.it; 8 Division of Epatobiliary Surgical Oncology, Istituto Nazionale Tumori IRCCS Fondazione Pascale—IRCCS di Napoli, 80131 Naples, Italy; f.izzo@istitutotumori.na.it

**Keywords:** machine learning, radiomic analysis, liver metastases, magnetic resonance imaging, tumor budding

## Abstract

Purpose: We aimed to assess the efficacy of machine learning and radiomics analysis using magnetic resonance imaging (MRI) with a hepatospecific contrast agent, in a pre-surgical setting, to predict tumor budding in liver metastases. Methods: Patients with MRI in a pre-surgical setting were retrospectively enrolled. Manual segmentation was made by means 3D Slicer image computing, and 851 radiomics features were extracted as median values using the PyRadiomics Python package. Balancing was performed and inter- and intraclass correlation coefficients were calculated to assess the between observer and within observer reproducibility of all radiomics extracted features. A Wilcoxon–Mann–Whitney nonparametric test and receiver operating characteristics (ROC) analysis were carried out. Balancing and feature selection procedures were performed. Linear and non-logistic regression models (LRM and NLRM) and different machine learning-based classifiers including decision tree (DT), k-nearest neighbor (KNN) and support vector machine (SVM) were considered. Results: The internal training set included 49 patients and 119 liver metastases. The validation cohort consisted of a total of 28 single lesion patients. The best single predictor to classify tumor budding was original_glcm_Idn obtained in the T1-W VIBE sequence arterial phase with an accuracy of 84%; wavelet_LLH_firstorder_10Percentile was obtained in the T1-W VIBE sequence portal phase with an accuracy of 92%; wavelet_HHL_glcm_MaximumProbability was obtained in the T1-W VIBE sequence hepatobiliary excretion phase with an accuracy of 88%; and wavelet_LLH_glcm_Imc1 was obtained in T2-W SPACE sequences with an accuracy of 88%. Considering the linear regression analysis, a statistically significant increase in accuracy to 96% was obtained using a linear weighted combination of 13 radiomic features extracted from the T1-W VIBE sequence arterial phase. Moreover, the best classifier was a KNN trained with the 13 radiomic features extracted from the arterial phase of the T1-W VIBE sequence, obtaining an accuracy of 95% and an AUC of 0.96. The validation set reached an accuracy of 94%, a sensitivity of 86% and a specificity of 95%. Conclusions: Machine learning and radiomics analysis are promising tools in predicting tumor budding. Considering the linear regression analysis, there was a statistically significant increase in accuracy to 96% using a weighted linear combination of 13 radiomics features extracted from the arterial phase compared to a single radiomics feature.

## 1. Introduction

Tumor budding, recognized as single cells or clusters of less than five cells, is considered as an aggressive histo-morphologic biomarker in cancer and has been well established as a poor prognostic feature in colorectal cancer, since high tumor budding is correlated with poor survival [1]. In colorectal cancer, tumor budding is associated with tumor progression and represents an additional prognostic factor in the TNM classification. Tumor buds can be found at the invasive front (peritumoral budding; PTB) and tumor center (intratumoral budding; ITB) of primary tumors [1,2]. The effects in patients with metastatic CRC (mCRC) were investigated. Previous studies have shown that tumor buds are also present in colorectal liver metastases (CRLM). In a meta-analysis, 1503 patients from nine retrospective cohort studies were evaluated and the authors demonstrated that, compared to those with low tumor budding, mCRC patients with high tumor budding are associated with poor progression-free survival, and therefore, they have a worse prognosis [2]. In addition, Noro et al. [3] assessed the rule of tumor budding in recurrences after hepatectomy in 52 patients with liver metastases, showing in a univariate analysis that preoperative chemotherapy, budding grade, extrahepatic metastases, and number of liver metastases at the time of recurrence were associated with overall survival (OS), while in a multivariate analysis, budding grade and number of liver metastases at the time of recurrence were associated with OS. The authors suggested that budding could be considered a new pathologic factor that affects the treatment choice [3]. Nowadays, tumor budding can only be assessed in surgical resection specimens, so that this prognostic marker has a limited value in patient risk evaluation in a pre-surgical setting. Radiomics analysis is an emerging field in research settings, since, thorough a mathematical approach, this allows us to obtain biological data from medical images [4,5,6,7,8]. Radiomics analysis allows multiple features to be obtained from medical imaging, including shape features and first-, second- or higher-order statistical features. After an adequate feature selection procedure to ensure the robustness of the parameters and eliminate redundant ones, these can be used as input predictors of machine learning methods in classification problems related to clinical oncological settings.

The great part of radiomic studies in an oncological setting is that they have a classification task or prediction of clinical outcomes as a target [9,10,11,12,13,14,15,16]. These approaches are guided by the idea that this analysis conveys data on tumor biology as a “virtual biopsy” that allows us to obtain information of the whole lesion and could be utilized more easily at multiple time points for disease evolution assessment [17,18,19,20,21,22].

Radiomics analysis could be a promising tool to “virtually” evaluate a lesion, with the possibility of analyzing the entire tumor during the history of the disease to obtain those markers that can influence the choice of treatment. Based on our knowledge, there are no studies in the literature that report the use of radiomics analysis in magnetic resonance for the evaluation of tumor budding.

The aim of this study is to evaluate the ability of machine learning and radiomics features, obtained from magnetic resonance images, to assess tumor budding in colorectal liver metastases patients.

## 2. Materials and Methods

### 2.1. Dataset Characteristics

The local ethics committee accepted this retrospective study waiving the signature of the patient’s consent due to the nature of the study.

The selection of patients was conducted from January 2018 to May 2021, considering the following inclusion criteria: (1) patients subjected to surgical resection for liver metastases; (2) proven pathological liver metastases; (3) patients subjected to MRI study in the pre-surgical setting with good-quality images; and (4) tumor budding assessment. Exclusion criteria were (1) no histological data, (2) no MRI studies, (3) low-quality MRI images and (4) no tumor budding assessment.

The patient cohort included a training set and an external validation set obtained from Careggi Hospital, Florence, Italy. A per lesion analysis was performed.

### 2.2. Imaging

A 1.5 T Magnetom Symphony scanner (Siemens, Erlangen, Germany) and a 1.5 T Magnetom Aera scanner (Siemens) equipped with an 8-element body and phased array coils were used for image acquisition of MR, including sequences obtained before and after intravenous (IV) injection of contrast medium. Volumetric interpolated T1-weighted SPAIR (VIBE) with controlled respiration was used to acquire images after IV injection of contrast agent (CA) with a liver-specific CA (0.1 mL/kg of Gd-EOB-BPTA, Primovist, Bayer Schering Pharma, Berlin, Germany). A power injector (Spectris Solaris^®^ EP MR, MEDRAD, Inc., Indianola, IA, USA) was used to deliver contrast agent at an infusion rate of 2 mL/s, and VIBE T1-w images were acquired in four different phases: arterial phase (35 s delay), portal venous phase (90 s), transition phase (120 s) and hepatobiliary excretion phase (20 min).

The study protocol is reported in Table 1.

### 2.3. Image Processing

**Figure 1:**
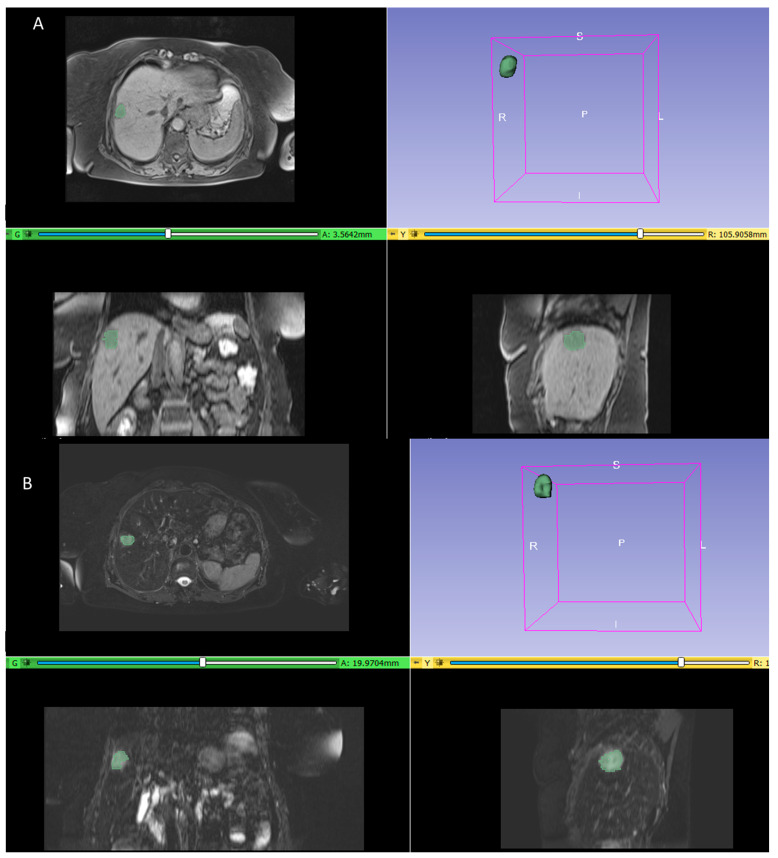
An example of segmentation step both on VIBE T1 weighted sequence (**A**) and on SPACE T2 weighted sequence images (**B**).

Two expert radiologists, with 20–25 years of experience in liver imaging, manually drew the contours of the lesions avoiding bias artifacts, slice by slice, on the arterial phase, on the portal phase, on the hepatobiliary excretion phase of the weighted VIBE sequence T1 and on the SPACE T2 weighted sequence, using the segmentation tools provided by 3D Slicer version 5.6.1 (available at the link https://download.slicer.org/ accessed on 15 January 2022). The radiologists performed the segmentation of the volumes of interest first separately and then in agreement with each other. Figure 1 shows an example of a segmentation phase.

Using PyRadiomics [https://pyradiomics.readthedocs.io/en/latest/features.html accessed on 15 January 2022], 851 radiomic features for each volume of interest were extracted as median values.

Radiomic characteristics are divided into first-order statistics; shape-based (3D); shape-based (2D); gray-level co-occurrence matrix; gray-level run-length matrix (16 features); gray-level zone size matrix (16 features); adjacent grayscale difference matrix (5 features); and gray-level dependency matrix. The radiomic characteristics are in accordance with the definitions of the Imaging Biomarker Standardization Initiative (IBSI). Descriptions are available at (https://readthedocs.org/projects/pyradiomics/downloads/ Data accessed 16 May 2021).

Radiomics analysis was performed blind to clinical and histopathological data on baseline images.

### 2.4. Statistical Analysis

The non-parametric Wilcoxon–Mann–Whitney test was performed to identify statistically significant differences in radiomics features between the two groups of patients with high-grade versus low-grade or no tumor budding.

Inter- and intraclass correlation coefficients (ICC) were calculated to evaluate the interobserver and intraobserver reproducibility of all radiomic features. Radiomic features with interclass and intraclass ICC > 0.75 were found to have good reproducibility and could be selected for model construction.

Balancing was performed through sample synthesis for underrepresented classes using the SASYNO (self-adaptive synthetic oversampling) approach. Using this procedure, balancing and increasing the number of cases in the population were carried out.

Receiver operating characteristic (ROC) analysis and Youden index were used to calculate the cut-off value to obtain the area under the ROC curve (AUC), sensitivity, positive predictive value (PPV), negative predictive value (NPV) and accuracy. The statistical significance of results for dichotomous tables was assessed using McNemar’s test.

In addition to the univariate analysis, multivariate analysis was performed to identify the combinations of the most significant radiomics features in classifying tumor budding. Significant features in the Wilcoxon–Mann–Whitney test with an intraclass ICC ≥ 0.75 and high accuracy greater than 75% were used as input in the least absolute selection and contraction operator (LASSO) method. At the end of the LASSO procedure, only the robust features were used in the classification phase. In the LASSO method, 10-fold cross-validation was used to select the optimal alpha smoothing parameter, since the mean square error of each patient was the smallest, and only parameters with a non-zero coefficient were reserved.

The linear regression model was used to evaluate the best linear combination of significant features, and classifiers based on machine learning methods were also adopted including support vector machine (SVM), k-nearest neighbors (KNN), artificial neural network (NNET) and decision tree (DT) as nonlinear methods. The best multivariate model was chosen considering maximum accuracy. Training was performed using 10,000-fold cross validation. Additionally, an external validation cohort was used to validate the results of the best classifier.

Statistics and Machine Toolbox of MATLAB R2021b (MathWorks, Natick, MA, USA) were used to perform all described statistical procedures.

A *p* value of ≤ 0.05 was considered significant.

## 3. Results

Forty-nine patients (18 women and 31 men) with a mean age of 60 years (range 36–82 years) and 119 liver metastases were included in the training set. The validation cohort, however, was composed of a total of 28 patients with a single lesion (9 women and 19 men) with an average age of 61 years (range 42–78 years).

Characteristics of patients and liver metastases are shown in Table 2.

In the univariate analysis (Table 3), the best predictors to classify the two groups of patients with high-grade versus low-grade or no tumor budding were as follows:

- original_glcm_Idn obtained in the T1-W VIBE sequence arterial phase with an accuracy of 84%, a sensitivity of 87% and a specificity of 77%;
- wavelet_LLH_firstorder_10Percentile obtained in the T1-W VIBE sequence portal phase with an accuracy of 92%, a sensitivity of 86% and a specificity of 81%;
- wavelet_HHL_glcm_MaximumProbability obtained in the T1-W VIBE sequence hepatobiliary excretion phase with an accuracy of 88%, a sensitivity of 94% and a specificity of 68%;
- wavelet_LLH_glcm_Imc1 obtained in the T2-W SPACE sequences with an accuracy of 88%, a sensitivity of 93% and a specificity of 71%.

All these findings were statistically significant in the McNemar test (*p* value ≤ 0.05).

**Figure 2:**
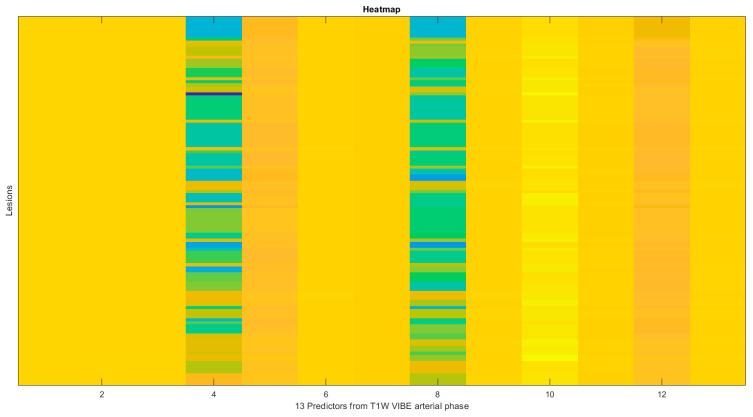
Heatmap of the 13 predictors from T1W VIBE arterial phase.

Considering the linear regression analysis (Table 4), to classify the two groups of patients with high-grade versus low-grade tumors or without tumor budding, there was a statistically significant increase in the accuracy to 96% (sensitivity of 99% and specificity of 87%, *p*-value < 0.05 in the McNemar test) using a weighted linear combination of 13 radiomic significant and robust features (Table 5) extracted from the arterial phase of the VIBE T1-W sequence (see Figure 2):

1. original_glcm_Idn;
2. original_glcm_Idm;
3. original_glcm_Id;
4. wavelet_LHH_firstorder_Minimum;
5. wavelet_LHH_firstorder_10Percentile;
6. wavelet_LLH_glcm_MaximumProbability;
7. wavelet_LLH_glcm_Imc1;
8. wavelet_LLH_firstorder_10Percentile;
9. wavelet_LLH_glrlm_GrayLevelNonUniformityNormalized;
10. wavelet_LLH_glrlm_LongRunEmphasis;
11. wavelet_LLH_glszm_SmallAreaLowGrayLevelEmphasis;
12. wavelet_HLH_firstorder_10Percentile;
13. wavelet_LLL_glcm_InverseVariance.

Considering a linear regression analysis of all significant data extracted from each MRI sequence, no increase in diagnostic performance in tumor budding classification was found.

**Figure 3:**
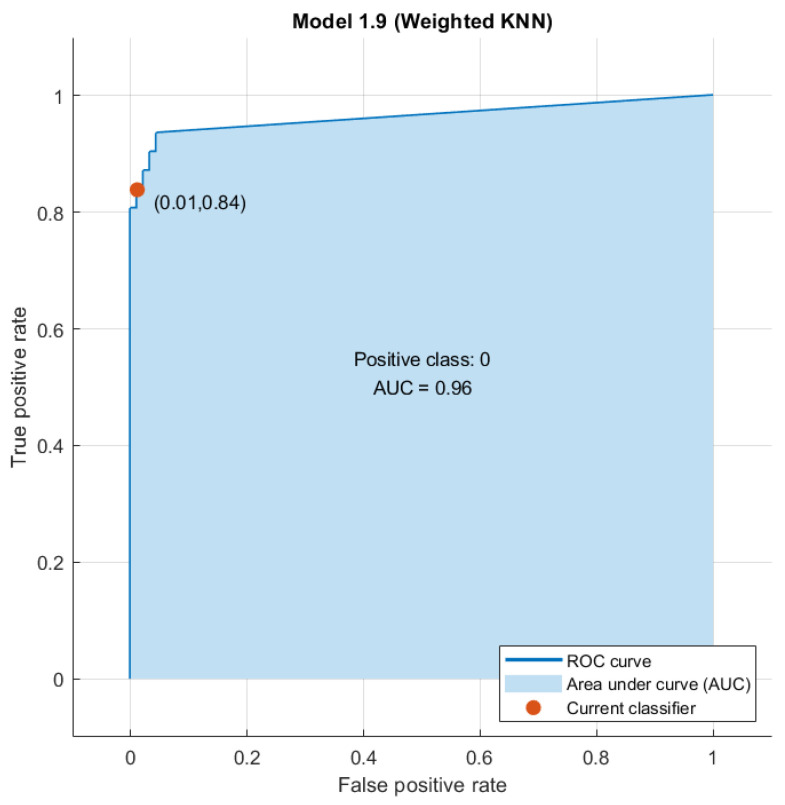
AUC of the best classifier (KNN) trained with the 13 radiomics features extracted from the arterial phase of the T1-W VIBE sequence.

Considering pattern recognition approaches in tumor budding classification, the best classifier was a KNN trained with the 13 radiomic features extracted from the arterial phase of the VIBE T1-W sequence, achieving 95% accuracy, 84% sensitivity, a specificity of 99% and an AUC of 0.96 (Figure 3). The validation set achieved an accuracy of 94%, a sensitivity of 86% and a specificity of 95%.

**Figure 4:**
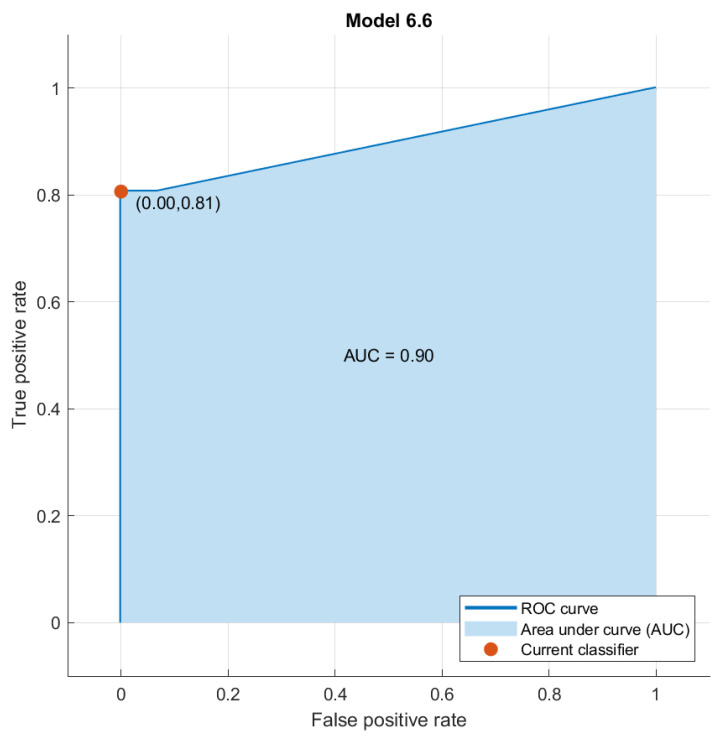
AUC of the best classifier (KNN) trained with all significant radiomic features extracted from each MRI sequence.

When we combined the significant features obtained from each MRI sequence, there was no increase in diagnostic performance in classifying tumor budding using pattern recognition approaches. However, the best classifier was a KNN which achieved an accuracy of 95%, a sensitivity of 100%, a specificity of 81% and an AUC of 0.90 (Figure 4).

## 4. Discussion

Tumor budding is recognized as a prognostic feature for primary colorectal cancer. In fact, although TNM classification remains the gold standard for prognostic stratification of colorectal cancer patients, heterogeneity in survival within the same stages required additional markers [23,24,25,26]. Several authors have found tumor budding to be independently associated with disease recurrence, cancer-related death and reduced overall survival (OS) [27,28,29,30,31,32,33,34,35]. In the setting of liver metastases, few data have been reported [36,37,38], and the main issue is that the only way to assess this pathological marker is in a surgical specimen. However, strong correlations between the KRAS/BRAF mutational status and tumor budding have been reported [36]. So, patients with liver metastases with tumor budding and/or KRAS mutational status [39,40] respond poorly to anti-EGFR therapy [32]. In the context of personalized medicine, this is evident as the possibility to predict several prognostic markers allows us to identify the best treatment for a specific patient [41,42,43,44,45,46]. Radiomics analysis could be a promising tool to evaluate a lesion “virtually”, with the possibility to analyze the whole tumor during the disease history to obtain those markers which can affect the treatment choice [47,48,49,50,51,52,53,54,55,56,57,58,59,60,61,62,63,64,65]. In addition, this approach is safe and inexpensive since radiomics data are obtained from radiological studies which a patient should be subjected during staging and follow-up [66,67,68,69,70,71,72,73,74,75,76,77,78,79,80,81,82,83,84].

Qu et al. [54], in a retrospective study on 266 patients, showed that radiomics analysis based on MR T2W sequences allowed us to predict tumor budding in patients with rectal cancer. To the best of our knowledge, only our group has assessed budding in liver metastases [4,8,85,86,87]. However, in a previous evaluation [85,86,87], we assessed specific phases of a contrast study. In this study, we evaluated the performance of all sequences performed during the study protocol. We have proven that in a univariate analysis, the best predictors to classify tumor budding were (a) original_glcm_Idn extracted in the T1-W VIBE sequence arterial phase with an accuracy of 84%, a sensitivity of 87% and a specificity of 77%; (b) wavelet_LLH_firstorder_10Percentile extracted in the T1-W VIBE sequence portal phase with an accuracy of 92%, a sensitivity of 86% and a specificity of 81%; (c) wavelet_HHL_glcm_MaximumProbability extracted in the T1-W VIBE sequence hepatobiliary excretion phase with an accuracy of 88%, a sensitivity of 94% and a specificity of 68%; and (d)wavelet_LLH_glcm_Imc1 extracted in the T2-W SPACE sequences with an accuracy of 88%, a sensitivity of 93% and a specificity of 71%. Analyzing these results, it is clear that all sequences should be assessed during radiomics evaluation. In addition, considering the linear regression analysis, a statistically significant increase in accuracy to 96% (sensitivity of 99% and a specificity of 87%) was obtained using a linear weighted combination of 13 radiomic features (original_glcm_Idn; original_glcm_Idm; original_glcm_Id; wavelet_LHH_firstorder_Minimum; wavelet_LHH_firstorder_10Percentile; wavelet_LLH_glcm_MaximumProbability; wavelet_LLH_glcm_Imc1; wavelet_LLH_firstorder_10Percentile; wavelet_LLH_glrlm_GrayLevelNonUniformityNormalized; wavelet_LLH_glrlm_LongRunEmphasis; wavelet_LLH_glszm_SmallAreaLowGrayLevelEmphasis; wavelet_HLH_firstorder_10Percentile; wavelet_LLL_glcm_InverseVariance) extracted from the arterial phase of the T1-W VIBE sequence. While considering a linear regression analysis of all significant features extracted in each MRI sequence, there was not an increase in diagnostic performance. With regard to the pattern recognition approaches, the best classifier is a KNN (settings: number of neighbors = 10; distance metric = Euclidean; distance weight = squared inverse; standardize data = true; hyperparameter options disabled) trained with the 13 radiomic features extracted from the arterial phase of the T1-W VIBE sequence, obtaining an accuracy of 95%, a sensitivity of 84%, a specificity of 99% and an AUC of 0.96. These data suggest that all contrast phases should be performed during follow-up of liver metastases.

Since previous studies demonstrated that mCRC patients with high tumor budding are associated with poor progression-free survival compared to those with low tumor budding, and therefore, have a worse prognosis [2], it has been suggested that budding could be considered a new pathologic factor that affects the treatment choice. However, nowadays, tumor budding can only be assessed in surgical resection specimens, so this prognostic marker has limited value in patient risk assessments in a pre-surgical setting. In this scenario, the possibility that a radiomics analysis allows us to obtain this feature, as we have demonstrated, may open up a new research method in the personalized medicine scenario.

Our results showed that radiomics is a promising tool to predict those markers that should be evaluated only on a surgical specimen. However, it is clear that there is a necessity to validate this approach, which is still in the research phase, considering the critical issues due to the lack of standardization, the quality of published studies, the low reproducibility, specially for MRI studies due to the high variability in the study protocol (e.g., scanners, sequences, contrast medium protocol), and the lack of standardization of the signal intensity (SI) [88]. Although MRI is the best modality to assess liver lesions [89,90,91,92,93,94,95,96,97,98,99,100,101,102,103,104,105,106,107,108,109], compared to computed tomography (CT), the variability in the SI assessment requires a normalization pre-processing phase [88], to increase the reproducibility of the results. However, this approach requires a multidisciplinary team (radiologists, biomedical engineers and medical physicists), which can only be found in a research center.

This study has the following limitations: (1) The small sample size, even if we assessed a homogeneous group, and it was a per lesion analysis; (2) the retrospective nature, which could cause selection bias; (3) a manual segmentation, which could cause interobserver variability; however, two expert radiologists in consensus approved this approach. Also, (4) we did not perform a normalization pre-processing approach, and finally, (5) we did not assess the chemotherapy effects; however, all patients were subjected to the same treatment, so this should not have affected our results. In addition, our results were validated by an external group to increase the study reproducibility.

## 5. Conclusions

Machine learning and radiomics analysis are promising tools in the prediction of tumor budding in liver metastases. All sequences and contrast phases should be performed since in the univariate analysis, the best predictors were obtained from the arterial phase, portal phase, hepatobiliary phase and T2-W SPACE sequences. In addition, considering the linear regression analysis, a statistically significant increase in accuracy to 96% (sensitivity of 99% and a specificity of 87%) was obtained using a linear weighted combination of 13 radiomic features extracted from the arterial phase of the T1-W VIBE sequence. Nowadays, tumor budding can only be assessed in surgical resection specimens, so this prognostic marker has limited value in patient risk assessments in a pre-surgical setting. In this scenario, the possibility that radiomics analysis allows us to obtain this feature may open up a new research method in the personalized medicine scenario.

## Figures and Tables

**Table 1:** Sequence parameters of MRI study protocol.

Sequence	Orientation	TR/TE/FA (ms/ms/deg.)	AT (min)	Acquisition Matrix	ST/Gap (mm)	FS
T2-W Trufisp	Coronal	4.30/2.15/80	0.46	512 × 512	4/0	Without
T2-W HASTE	Axial	1500/90/170	0.36	320 × 320	5/0	Without and with (SPAIR)
T2W HASTE	Coronal	1500/92/170	0.38	320 × 320	5/0	Without
T2W SPACE	Axial	4471/259/120	4.20	384 × 450	3/0	With (SPAIR)
T1-W In-Out phase	Axial	160/2.35/70	0.33	256 × 192	5/0	Without
DWI	Axial	7500/91/90	7	192 × 192	3/0	Without
T1-W VIBE	Axial	4.80/1.76/30	0.18	320 × 260	3/0	With (SPAIR)

Note. W = weighted, TR = repetition time, TE = echo time, FA = flip angle, AT = acquisition time, SPAIR = Spectral Adiabatic Inversion Recovery, HASTE = Half-Fourier Single-Shot Turbo Spin-Echo, VIBE = volumetric interpolated breath hold examination.

**Table 2:** Characteristics of the study population (77 patients and 147 metastases) including both internal and external validation datasets.

Patient Description	Numbers (%)/Range
Sex	Men 50 (64.9%)
	Women 27 (35.1%)
Age	61 years; range: 36–82 years
Primary cancer site	
Colon	52 (67.5%)
Rectum	25 (32.5%)
Hepatic metastases description	147
Patients with single nodule	48 (62.3%)
Patients with multiple nodules	29 (37.7%)/range: 2–13 metastases
Nodule size (mm)	median size 35.8 mm; range 7–58 mm
Tumor budding	
Absent	19 (13%)
Low grade	18 (12%)
High grade	110 (75%)

**Table 3:** Diagnostic performance in the univariate analysis in the classification of the two groups of patients with high-grade versus low-grade or no tumor budding.

Diagnostic Performance	T1-W VIBE Sequence Arterial Phase	T1-W VIBE Sequence Portal Phase	T1-W VIBE Sequence Hepatobiliary Excretion Phase	T2-W SPACE
	original_glcm_Idn	wavelet_LLH_firstorder_10Percentile	wavelet_HHL_glcm_MaximumProbability	wavelet_LLH_glcm_Imc1
AUC	0.74	0.80	0.70	0.77
Sensitivity	0.87	0.96	0.94	0.93
Specificity	0.77	0.81	0.68	0.71
PPV	0.92	0.93	0.89	0.90
NPV	0.67	0.86	0.81	0.79
Accuracy	0.84	0.92	0.88	0.88
Cut-off	0.94	−37.14	0.28	−0.14

**Table 4:** Diagnostic performance in the linear regression analysis in the classification of the two groups of patients with high-grade versus low-grade or no tumor budding.

Diagnostic Performance	T1-W VIBE Sequence Arterial Phase	T1-W VIBE Sequence Portal Phase	T1-W VIBE Sequence Hepatobiliary Excretion Phase	T2-W SPACE
	Linear Regression Model of	wavelet_LLH_firstorder_10Percentile	wavelet_HHL_glcm_MaximumProbability	wavelet_LLH_glcm_Imc1
AUC	0.90	0.89	0.81	0.89
Sensitivity	0.99	1.00	0.89	0.92
Specificity	0.87	0.87	0.84	0.94
PPV	0.96	0.96	0.94	0.98
NPV	0.96	1.00	0.72	0.81
Accuracy	0.96	0.96	0.88	0.93
Cut-off	0.49	0.59	0.67	0.65

**Table 5:** The best linear regression model.

Variables	Coefficients
Intercept	−6.88
original_glcm_Idn	21.37
original_glcm_Idm	47.83
original_glcm_Id	−56.56
wavelet_LHH_firstorder_Minimum	0.01
wavelet_LHH_firstorder_10Percentile	−0.03
wavelet_LLH_glcm_MaximumProbability	2.02
wavelet_LLH_glcm_Imc1	9.51
wavelet_LLH_firstorder_10Percentile	−0.01
wavelet_LLH_glrlm_GrayLevelNonUniformityNormalized	−3.54
wavelet_LLH_glrlm_LongRunEmphasis	−0.01
wavelet_LLH_glszm_SmallAreaLowGrayLevelEmphasis	2.52
wavelet_HLH_firstorder_10Percentile	0.27
wavelet_LLL_glcm_InverseVariance	−5.27

## Data Availability

Data are available at link https://zenodo.org/records/10464602 accessed on 6 January 2023.
